# A symbiotic mosquito‐gut bacterium for flavivirus control

**DOI:** 10.1002/ctm2.70087

**Published:** 2024-11-29

**Authors:** Jinglin Wang, Yibin Zhu, Jun Leng, Yulin Yuan, Yingyi Cao, Gong Cheng, Xueshan Xia

**Affiliations:** ^1^ Yunnan Key Laboratory of Cross‐Border Infectious Disease Controlling and Novel Drug Development (Under construction) & Yunnan Provincial Key Laboratory of Public Health and Biosafety School of Public Health, Kunming Medical University Kunming China; ^2^ New Cornerstone Science Laboratory, Tsinghua University‐Peking University Joint Center for Life Sciences School of Basic Medical Sciences, Tsinghua University Beijing China; ^3^ Shenzhen Bay Laboratory Institute of Infectious Diseases Shenzhen China; ^4^ Southwest United Graduate School Kunming China

**Keywords:** control, dengue virus, symbiotic mosquito‐gut bacterium, Zika virus

## Abstract

*Dengue virus* (DENV) and *Zika virus* (ZIKV) have emerged as major global public health challenges, causing numerous infections and fatalities each year. However, current measures, including vaccines and treatments, are often limited or ineffective. This highlights the urgent need for novel preventive strategies to control the spread of key mosquito‐borne viruses like DENV and ZIKV. In a recent study published in Science, Zhang et al. isolated a bacterium named *Rosenbergiella*_YN46 from the gut of field‐caught Aedes albopictus mosquitoes in Yunnan Province, China. This commentary reviews their findings, published on April 19, 2024, which describe the symbiotic bacterium Rosenbergiella_YN46 and its ability to block flavivirus transmission, including both DENV and ZIKV. The bacterium shows promising potential for future dengue fever prevention and provides valuable insights into a novel biological approach for controlling mosquito‐borne viral diseases.

## Graphical abstract

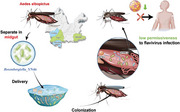

1

Mosquito‐borne viruses have become a global public health challenge, causing numerous infections and deaths each year.^1^ *Aedes albopictus* and *Aedes aegypti* are highly invasive species and serve as the primary vectors for several arboviruses, including dengue virus (DENV), chikungunya virus and Zika virus (ZIKV).^2^ As a member of the Flavivirus genus, DENV is widespread in tropical and subtropical regions, often causing periodic outbreaks. The simultaneous transmission of different DENV serotypes increases the severity of dengue and elevates the risk of epidemic outbreaks. In recent years, another flavivirus, ZIKV, has attracted attention due to its association with neurological complications, such as microcephaly.^3^ The potential for new mosquito‐borne disease outbreaks remains a constant threat. Unfortunately, existing measures against these diseases, including vaccines and medications, are often unavailable or ineffective. Therefore, there is an urgent need for new preventive strategies to control the spread of key mosquito‐borne viruses such as DENV and ZIKV (See Figure 1)

**FIGURE 1:**
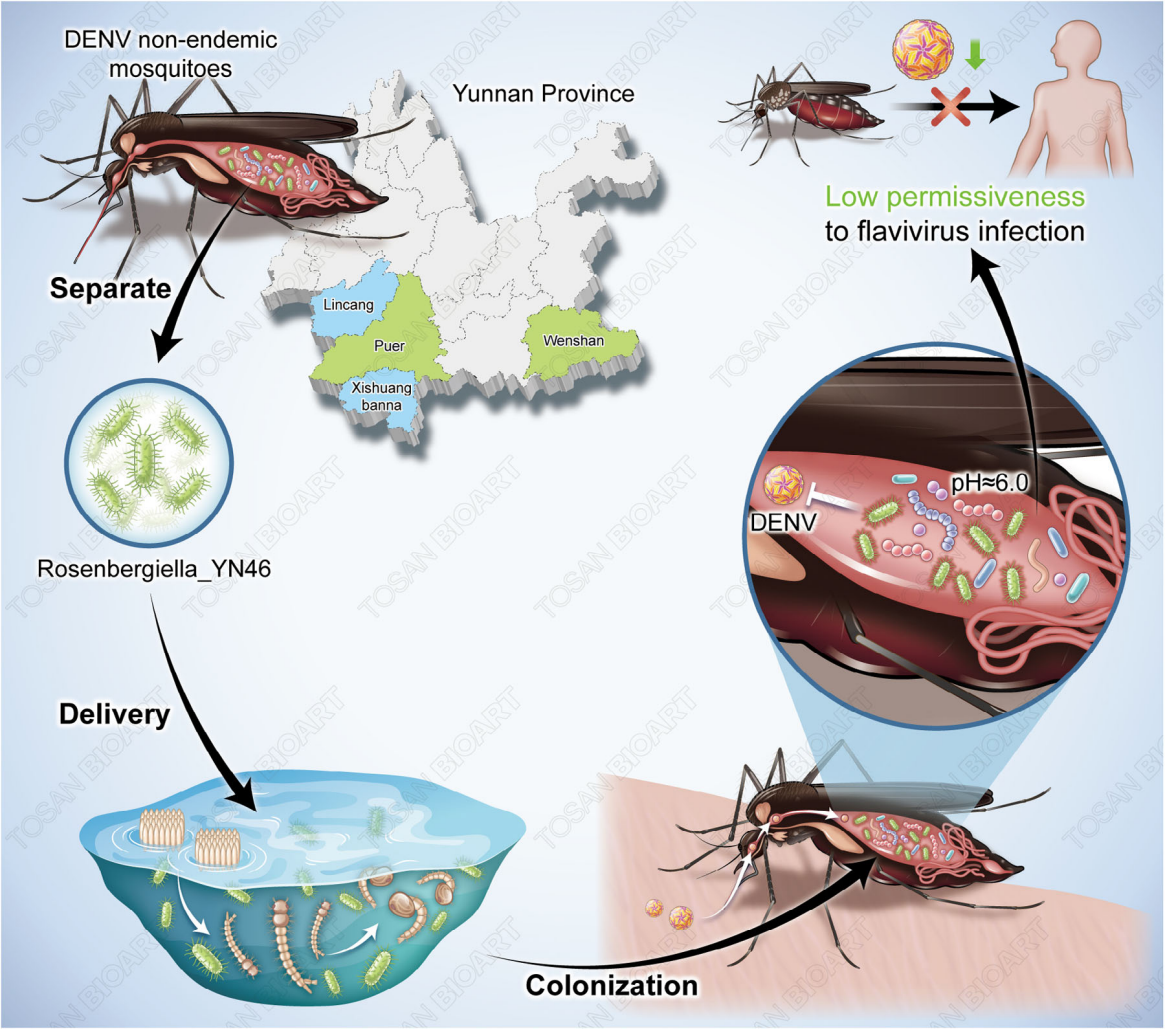
*Rosenbergiella*_YN46 in field isolates of A*. albopictus* and Semifield intervention with *Rosenbergiella* _YN46 reduces DENV in *A. albopictus*.

A growing number of researches have demonstrated that the rich microbiota in mosquitoes plays a complex role in the transmission of these viruses. Notably, the gut microbiota of mosquitoes is considered a key factor in flavivirus infection and transmission.^4^ In a recent study published in *Science*, Zhang et al. isolated a bacterium named *Rosenbergiella*_YN46 from the gut of field‐caught *Aedes albopictus* mosquitoes in Yunnan Province, China.^5^ This study demonstrated the correlation between the colonization of this bacterium in the mosquito gut and the infectivity of DENV and ZIKV, offering promising strategies for controlling these diseases.

The researchers conducted an epidemiological analysis of dengue outbreaks in Yunnan Province in the past decade and found significant geographic heterogeneity in dengue incidence. The team examined mosquitoes from four cities in southern Yunnan with similar environmental conditions but differing levels of dengue incidence. Annual dengue outbreaks were reported in Xishuangbanna and Lincang, while few local cases were reported in neighbouring Wenshan and Pu'er. To explore the reasons behind this geographical discrepancy, the researchers collected *Aedes albopictus* mosquitoes from these four regions and analyzed the 16S rRNA sequences of the mosquito gut bacteria. They found that *Rosenbergiella*_YN46 was highly abundant in the gut of mosquitoes from Wenshan and Pu'er (non‐dengue‐endemic areas) but had lower abundance in the gut of mosquitoes from Xishuangbanna and Lincang (dengue‐endemic areas), suggesting a strong negative correlation between the natural distribution of *Rosenbergiella*_YN46 and dengue incidence.

By introducing *Rosenbergiella*_YN46 into the mosquito gut, Zhang *et al*. found that mosquitoes carrying this bacterium were 3.3 to 5.2 times less likely to be infected with DENV2 and ZIKV.^6^ These findings suggest that *Rosenbergiella*_YN46 confers resistance to flavivirus infection, likely through its unknown components. The researchers identified 10 proteins derived from *Rosenbergiella*_YN46 through mass spectrometry and tested them. They discovered that this bacterium secretes glucose dehydrogenase (*Ry*GDH), which converts glucose into gluconolactone, and this then hydrolyzes into gluconic acid, lowering the pH of the mosquito gut.^7^ By analyzing the relationship between gut pH and the abundance of *Rosenbergiella*_YN46, they found that the mosquito gut's pH level negatively correlated with the bacterium's abundance. Further in vitro experiments demonstrated *Ry*GDH's acidification ability, and blood‐feeding infection assays on mosquitoes carrying *Rosenbergiella*_YN46 revealed that the mosquito gut's pH dropped significantly to around 6.0, confirming that *Rosenbergiella*_YN46 acidifies the mosquito gut, inactivating the flavivirus E protein and preventing the virus from entering cells.

To test the practical applicability of these findings, the researchers explored the effectiveness of environmental interventions with *Rosenbergiella*_YN46 in blocking virus infection and transmission. They conducted laboratory experiments by hatching wild *Aedes albopictus* mosquito eggs collected from Xishuangbanna in water containing *Rosenbergiella*_YN46 and examined the colonization of the bacterium in the larvae, pupae and adult mosquitoes. High colonization rates were found in all stages. Additionally, they set up a semi‐field intervention experiment in Mengla County, Xishuangbanna, using a “mosquito sphere” to simulate natural conditions. Mosquito larvae were hatched in water containing *Rosenbergiella*_YN46, and the researchers observed high colonization rates in the mosquito gut and significantly lower infection rates with DENV2 in mosquitoes treated with the bacterium. These results demonstrate that *Rosenbergiella*_YN46 can colonize *Aedes* mosquitoes throughout their life cycle and significantly reduce their infection with DENV2.

In recent years, the use of mosquito microbiota has shown great potential in reducing the transmission of pathogens by mosquitoes. The microbiota plays a vital role in many biological processes in mosquitoes, including nutrition, digestion, mating, reproduction, development, immune responses and pathogen resistance.^8^ In the study by Zhang et al., a symbiotic bacterium, *Rosenbergiella*_YN46, was isolated from non‐endemic regions in Yunnan Province. As an environmentally friendly nectar‐associated bacterium, *Rosenbergiella*_YN46 can colonize the gut of *Aedes aegypti* and *Aedes albopictus*, and it inhibits flavivirus infection by secreting glucose dehydrogenase (*Ry*GDH), which acidifies the mosquito gut environment. This biocontrol strategy could potentially reduce the transmission of mosquito‐borne diseases like dengue fever, offering a more environmentally sustainable approach compared to traditional chemical insecticides.

## AUTHOR CONTRIBUTIONS

Conceptualization, Gong Cheng and Xueshan Xia; Writingreview and editing, Jinglin Wang, Yibin Zhu, Jun Leng, Yulin Yuan, Yingyi Cao. All authors have read and agreed to the published version of the manuscript.

## CONFLICT OF INTEREST STATEMENT

The authors declare no conflict of interest.

## ETHICS STATEMENT

This study was conducted in compliance with data protection regulations.The use of personal data was approved by the Kunming Medical University Ethics Committee, ensuring that all data was anonymized and securely stored.

## References

[1] ZHU Y , LIU J , CHENG G . Progress towards research on mosquito‐borne arboviral transmission and infection. Sci Bull. 2023;68(23):2884‐2888. 10.1016/j.scib.2023.10.040 37940452

[2] ZHANG L , WANG D , SHI P , et al. A naturally isolated symbiotic bacterium suppresses flavivirus transmission by Aedes mosquitoes. Science. 2024;384(6693):eadn9524. 38669573 10.1126/science.adn9524

[3] YUE Y , LIU Q , LIU X , et al. Comparative analyses on epidemiological characteristics of dengue fever in Guangdong and Yunnan, China, 2004–2018. BMC Pub Health. 2021;21(1):1389. 34256730 10.1186/s12889-021-11323-5 PMC8278621

[4] ZHU Y , CHENG G . Making hosts smell tastier: how mosquito‐borne viruses take the initiative in viral transmission. Clin Transl Med. 2023;13(1):e1168. 36610034 10.1002/ctm2.1168 PMC9825108

[5] SHI H , YU X , CHENG G . Impact of the microbiome on mosquito‐borne diseases. Protein Cell. 2023;14(10):743‐761. 37186167 10.1093/procel/pwad021 PMC10599646

[6] SCOLARI F , SANDIONIGI A , CARLASSARA M , et al. Exploring changes in the microbiota of *Aedes albopictus*: comparison among breeding site water, larvae, and adults. Front Microbiol. 2021;12:624170. 33584626 10.3389/fmicb.2021.624170 PMC7876458

[7] KRAEMER MU , SINKA ME , DUDA KA , et al. The global distribution of the arbovirus vectors *Aedes aegypti* and Ae. albopictus. Elife. 2015;4:e08347. 26126267 10.7554/eLife.08347 PMC4493616

[8] BING XL , LIANG ZJ , TIAN J , et al. The influence of Acetobacter pomorum bacteria on the developmental progression of Drosophila suzukii via gluconic acid secretion. Mol Ecol. 2024;33(2):e17202. 37947376 10.1111/mec.17202

